# Clinical Performance of Bulk-Fill Versus Incremental Composite Restorations in Primary Teeth: A Systematic Review of In Vivo Evidence

**DOI:** 10.3390/dj13070320

**Published:** 2025-07-15

**Authors:** Maria Sarapultseva, Desheng Hu, Alexey Sarapultsev

**Affiliations:** 1 Department of Pediatric Dentistry, Medical Firm Vital EVV, Ekaterinburg 620144, Russia; 2 Department of Integrated Traditional Chinese and Western Medicine, Union Hospital, Tongji Medical College, Huazhong University of Science and Technology, Wuhan 430022, China; desheng.hu@hust.edu.cn; 3 Hubei Key Laboratory of Biological Targeted Therapy, Union Hospital, Tongji Medical College, Huazhong University of Science and Technology, Wuhan 430022, China; 4 China-Russia Medical Research Center for Stress Immunology, Union Hospital, Tongji Medical College, Huazhong University of Science and Technology, Wuhan 430022, China; 5 Russian-Chinese Education and Research Center of System Pathology, South Ural State University, 76 Lenin Prospekt, Chelyabinsk 454080, Russia; 6 Institute of Immunology and Physiology, Ural Branch of the Russian Academy of Science, 106 Pervomaiskaya Street, Ekaterinburg 620049, Russia

**Keywords:** bulk-fill composite, pediatric dentistry, primary teeth, restoration, systematic review, retention, survival, marginal integrity

## Abstract

**Background and Objectives**: This is the first systematic review to focus exclusively on in vivo randomized controlled trials that compare bulk-fill and conventional incremental composite restorations in primary teeth. Our aim was to synthesize current evidence on their clinical performance, including retention, two-year survival rates, marginal integrity, and procedural efficiency. **Methods**: A comprehensive literature search was conducted in PubMed, Scopus, and the Elicit AI platform up to March 2025. Eligible studies were in vivo randomized controlled trials involving children aged 3–12 years with carious primary teeth, directly comparing bulk-fill and incremental composite restorations. Primary outcomes included retention rates, two-year survival, and marginal integrity, while secondary outcomes were postoperative sensitivity, secondary caries, and aesthetic outcomes. Two reviewers independently performed study selection, data extraction, and risk-of-bias assessments using the Cochrane RoB 2.0 tool. A narrative synthesis was undertaken due to substantial heterogeneity in study design and outcome reporting. The review protocol was registered in PROSPERO (CRD420251021433). **Results**: Thirteen randomized controlled trials met the inclusion criteria. Both restoration techniques demonstrated high short-term retention rates (>90%) and comparable two-year survival (85–90%). Marginal integrity was generally equivalent, though incremental techniques showed modest advantages in complex cavities. Secondary outcomes were inconsistently reported, with no significant group differences. Bulk-fill restorations consistently reduced the procedural time by 2–4 min per restoration, representing a meaningful advantage in pediatric clinical settings. **Conclusions**: Bulk-fill composites offer a clinically effective and time-efficient alternative to incremental layering in the restoration of primary teeth. This focused synthesis addresses a gap in existing reviews by concentrating solely on primary dentition and in vivo evidence. Despite similar clinical outcomes, the time savings associated with bulk-fill techniques may enhance their utility in pediatric dentistry. Further standardized and long-term trials are warranted to confirm these findings and inform clinical guidelines.

## 1. Introduction

Dental caries is among the most prevalent chronic diseases affecting children worldwide, necessitating effective restorative treatments to preserve function and aesthetics in primary teeth. Resin composites are widely regarded as the material of choice for direct restorations due to their favorable mechanical properties and aesthetic outcomes [1,2]. Traditionally, incremental layering techniques have been employed to minimize polymerization shrinkage stress and ensure optimal marginal adaptation [2]. However, these methods are time-intensive and technique-sensitive, posing challenges in pediatric dentistry where patient cooperation and efficiency are critical [3].

Bulk-fill resin composites represent a significant advancement in restorative materials, offering simplified placement techniques that allow single-layer application up to 4–5 mm thickness [4]. These materials feature enhanced translucency, improved photoinitiators, and polymerization modulators to achieve deeper curing depths and reduced shrinkage stress [1,2,5]. In clinical practice, bulk-fill composites have demonstrated comparable survival rates and retention performance to conventional incremental composites in both permanent and primary teeth restorations [2,6]. Furthermore, their reduced placement time has been shown to improve efficiency, which is particularly advantageous in pediatric settings [5].

Despite these promising attributes, the clinical performance of bulk-fill composites in primary teeth remains under investigation. Reported outcomes—such as marginal adaptation, secondary caries, and postoperative sensitivity—are similar, but long-term durability and cavity-specific performance vary [5,7,8,9]. Moreover, differences in adhesive systems, cavity design, and material formulations may influence the success of restorations.

Several gaps remain unaddressed. Few studies extend beyond 12 months, despite the importance of 2-year survival rates for evaluating restoration longevity in primary dentition. There is inconsistency in how studies define and assess retention, survival, and marginal integrity, including the use (or absence) of validated scoring systems, such as USPHS or FDI. This variability complicates direct comparison and pooled analysis. Important clinical indicators—such as postoperative sensitivity, secondary caries, and aesthetic performance—are often inadequately reported or omitted, despite their relevance for clinical decision-making. Elements such as adhesive strategy, isolation method, cavity classification, and bulk-fill material viscosity are seldom analyzed in relation to outcomes, even though they may significantly affect restoration success. While bulk-fill composites are promoted for their time-saving advantages, evidence regarding their clinical superiority or non-inferiority compared to conventional incremental techniques remains mixed and inconclusive.

The present systematic review was conducted in accordance with the PRISMA 2020 statement [10] and is based on a pre-registered protocol (PROSPERO ID: CRD42023471088). Its primary aim is to evaluate the comparative efficacy of bulk-fill versus conventional incremental composite restorations in primary teeth using well-defined clinical performance metrics from in vivo studies. Specifically, it synthesizes evidence on retention rates, survival outcomes, and marginal integrity, while also considering secondary endpoints, such as postoperative sensitivity, aesthetic results, and secondary caries incidence.

The review question was formulated using the PICO framework, as follows: in children with carious primary teeth (P), do bulk-fill composite restorations (I), compared to conventional incremental composite restorations (C), provide superior or equivalent outcomes in terms of retention, two-year survival, and marginal integrity (O)?

By clarifying the clinical effectiveness of these restorative approaches, this review aims to support evidence-based decision-making in pediatric dentistry and contribute to the refinement of restorative protocols.

## 2. Materials and Methods

This systematic review was conducted between January and March 2025, following a pre-specified protocol in accordance with PRISMA 2020 guidelines. The protocol was registered in the PROSPERO database (CRD420251021433) and is available at: https://www.crd.york.ac.uk/prospero/display_record.php?ID=CRD420251021433 (accessed on 31 March 2025). No amendments were made after registration.

### 2.1. Eligibility Criteria

Studies were included if they enrolled pediatric patients (aged 3–12 years) with carious primary teeth, compared bulk-fill resin composites to conventional incremental layering techniques, and reported at least one primary clinical outcome—namely, restoration retention, two-year survival, or marginal integrity. Secondary outcomes of interest included postoperative sensitivity, color match or other aesthetic parameters, surface texture, anatomical form, and the incidence of secondary caries. Eligible study designs comprised in vivo randomized controlled trials (RCTs), prospective clinical trials, and retrospective clinical studies. Only full-text, peer-reviewed articles published in English were considered.

During full-text screening, studies were excluded based on a priori PICO framework criteria and methodological quality considerations. Exclusion criteria included studies that did not involve restorations in primary teeth, lacked pediatric participants, or used non-human models. Studies were also excluded if they failed to report the type of restorative material used, did not compare bulk-fill with conventional incremental composites, or lacked a well-defined control group. Additional exclusions were made for studies that did not report at least one of the key clinical outcomes (retention, survival, or marginal integrity), as well as for investigations that focused solely on surrogate markers, in vitro performance, or laboratory outcomes. Studies with undefined follow-up periods, or with less than 12 months of clinical observation, were not considered. Furthermore, studies with insufficient methodological detail, poor reporting quality, or missing essential data—such as sample size, baseline characteristics, or restoration protocol—were excluded. Abstract-only publications, duplicates, and studies for which the full text could not be retrieved despite extensive efforts were also excluded. No automation tools were used during screening or eligibility assessment. All decisions were made by human reviewers.

### 2.2. Information Sources and Search Strategy

A comprehensive literature search was conducted on 25 March 2025 across three sources: PubMed, Scopus, and the Elicit AI platform. The search strategy combined both MeSH terms and free-text keywords related to composite restorations, pediatric dentistry, and clinical outcomes. The detailed database-specific search strings, including Boolean operators, filters, and syntax, are provided in Supplementary S1. No age restrictions were applied during database querying in order to ensure inclusion of all potentially relevant studies involving primary dentition. In addition to electronic searches, reference lists of included studies were manually screened to identify additional eligible records.

### 2.3. Study Selection

**Figure 1:**
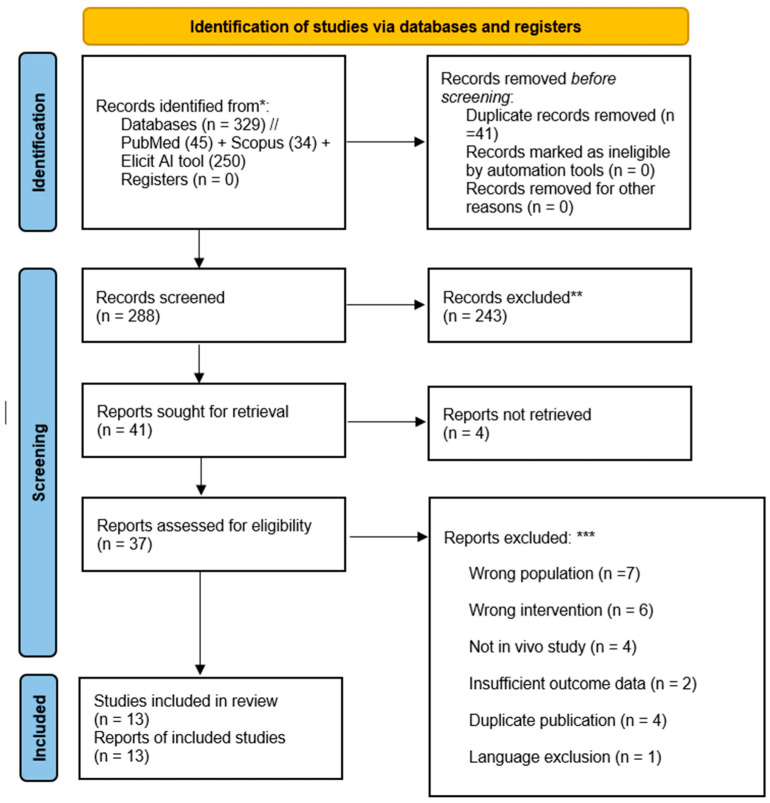
PRISMA 2020 flow diagram. Here, * 250 entries were screened using the Elicit AI tool (AI-based literature retrieval system with no explicit database specification). Relevant records from Elicit were independently verified and duplicates were removed before screening. ** No records were excluded using automation tools—all exclusions were performed manually by human reviewers. *** A complete list of excluded studies with specific reasons is available upon request.

A total of 329 records were identified through searches of PubMed (45), Scopus (34), and the Elicit AI tool (250). After removing 41 duplicates, 288 unique records remained and were screened by title and abstract according to predefined eligibility criteria. Following this process, full texts of potentially relevant studies were assessed in detail, and 13 articles were finalized for inclusion in the systematic review. The complete screening and selection process is illustrated in the PRISMA 2020 flow diagram (Figure 1), and a list of excluded studies with specific reasons for exclusion is available upon request.

### 2.4. Data Extraction

Data extraction was conducted using a customized form in the Elicit platform, designed to capture key study parameters, including design type, location, sample size, participant age, cavity classification, restorative technique (bulk-fill vs. incremental), follow-up duration, and evaluation criteria (e.g., USPHS and FDI). Extracted outcomes included restoration retention, survival rate at defined intervals, marginal adaptation, and secondary measures, such as postoperative sensitivity, aesthetic performance, and incidence of secondary caries. Information on adhesive systems, isolation techniques, operator experience, and funding sources was also collected when available. When study reports lacked clarity on specific variables, context-based assumptions were made (e.g., standard use of universal adhesives), and authors were contacted for clarification when necessary. While inter-rater reliability metrics (e.g., Cohen’s kappa) were not formally calculated, all discrepancies were resolved through discussion and consensus between the two reviewers.

### 2.5. Risk of Bias Assessment

Risk of bias was assessed independently by two reviewers using the Cochrane Risk of Bias 2.0 (RoB 2) tool, which evaluates five domains: the randomization process, deviations from intended interventions, missing outcome data, outcome measurement, and selective reporting. Because all included studies were RCTs, ROBINS-I was not used. Discrepancies in risk assessment were resolved through consensus discussion.

### 2.6. Data Synthesis

Due to substantial heterogeneity across studies in terms of outcome definitions, scoring systems (USPHS, FDI, or modified), restorative protocols, and follow-up durations, a formal meta-analysis was not performed. Instead, a narrative synthesis was conducted, stratifying findings by intervention type and clinical outcomes. For the primary outcomes, the effect measures included the percentage of restorations classified as clinically successful according to study-specific criteria. For secondary outcomes, both binary (e.g., presence or absence of secondary caries) and proportion-based data (e.g., Alpha scores) were reported descriptively. No data transformation or imputation was undertaken

### 2.7. Assessment of Reporting Bias and Certainty of Evidence

The certainty of the body of evidence was rated using the GRADE approach, which considers study limitations, inconsistency, indirectness, imprecision, and risk of publication bias. Funnel plot asymmetry was visually assessed for outcomes reported in ≥10 studies, although the low number of eligible studies limited interpretability. GRADE assessments were performed narratively without pooled estimates, in line with current recommendations for qualitative syntheses.

## 3. Results

All 13 included studies were randomized controlled trials (RCTs) [8,11,12,13,14,15,16,17,18,19,20,21,22] (Table 1). According to the Cochrane RoB 2.0 tool, the overall risk of bias was judged to be low to moderate, with no studies rated as high risk (Table S1). Specifically:

- Five studies were assessed as having low risk of bias across all domains [11,12,15,20,21].
- The remaining studies showed “some concerns” in one or more domains, most frequently in the randomization process (due to incomplete reporting on allocation procedures) and measurement of the outcome.
- One study had multiple domains with “some concerns” (randomization, outcome measurement, and selective reporting) but was not judged to have high risk in any domain [14].

**Table 1:** Characteristics of the included studies.

Study	Study Design	Sample Size	Follow-Up Period	Evaluation Criteria	Population Details	Intervention Materials
Akman and Tosun, 2020 [11]	RCT, Prospective	30 patients, 160 restorations	12 months	Modified USPHS	Children aged 6–10, primary molars	Sonicfill, X-tra fil vs. Filtek Z550
Banon et al., 2024 [12]	RCT, Split-mouth	20 children, 96 molars	24 months	USPHS-Ryge	Children aged 5–10, primary molars	ACTIVA BioACTIVE vs. Dyract eXtra
Deepika et al., 2022 [13]	RCT, Split-mouth	50 children, 100 primary molars	12 months	Modified USPHS	Children aged 5–9, primary molars	ACTIVA Bioactive vs. Beautifil flow Plus
Ehlers et al., 2019 [14]	RCT, Split-mouth	32 children	12 months	FDI	Children aged 4–9, primary molars	Venus Bulk Fill vs. Dyract eXtra
Gindri et al., 2022 [15]	RCT	65 participants, 140 restorations	12 months	FDI	Children aged 5.2–8.2, primary molars	Filtek Bulk Fill vs. Filtek Z350 XT
Cantekin and Gumus, 2014 [16]	RCT, Prospective	20 children	12 months	Modified Zurn and Seale	Children aged 5–7, primary molars	SDR flow vs. Aelite LS Posterior
Lardani et al., 2022 [17]	Split-mouth RCT	45 children	12 months	FDI	Children aged 5–9, primary molars	ACTIVA BioActive, SDR Bulk-fill
Lucchi et al., 2024 [18]	Retrospective study	198 patients	5 years	USPHS	Children aged 0–12, 673 restorations	Filtek Bulk-Fill Flow
Massa et al., 2022 [19]	RCT	62 subjects, 144 primary molars	18 months	FDI	Children aged 4.2–7.6, primary molars	Filtek Bulk Fill Posterior
Olegário et al., 2022 (1) [20]	RCT	91 children	12 months	Roeleveld	Children aged 3–8, primary molars	Filtek Bulk Fill
Olegário et al., 2022 (2) [21]	RCT	93 children	24 months	Roeleveld	Children aged 4–8, primary molars	Filtek Bulk Fill
Sarapultseva and Sarapultsev, 2019 [22]	Split-mouth RCT	27 children	24 months	Modified Ryge	Children aged 3–6, mandibular molars	SDR vs. Ceram-X mono
Öter et al. [8]	RCT, Split-mouth	80 children	12 months	Modified USPHS	Children aged 5.61–9.21, primary molars	Filtek Bulk-Fill vs. Filtek Z250

There were no major concerns regarding missing outcome data, and most studies used standardized assessment tools (e.g., USPHS or FDI criteria), enhancing the credibility of reported outcomes. These findings support a moderate-to-high level of confidence in the synthesized evidence, while still encouraging cautious interpretation due to variability in trial conduct and reporting rigor.

### 3.1. Study Characteristics and Overview

Because the studies differed too much to allow for a meta-analysis, we summarized the results narratively and grouped them by outcome.

The 13 included studies, published between 2014 and 2024, involved pediatric patients ranging in age from approximately 3 to 13 years [8,11,12,13,14,15,16,17,18,19,20,21,22] (Table 1). Sample sizes varied from 20 to 198 children (Table S1). The study designs included both parallel-group and split-mouth designs, with follow-up durations ranging from 12 to 60 months—most commonly reporting outcomes at 12 or 24 months. Across all studies, bulk-fill composite restorations were compared against conventional incremental layering techniques. Bulk-fill materials were typically placed in single increments of up to 4–5 mm, while conventional composites were applied in 2 mm layers. Most studies used high-viscosity bulk-fill resins and evaluated clinical performance using standardized scoring systems, such as USPHS or FDI criteria (Table 1).

Outcomes assessed included:

- Primary outcomes: retention, survival rate at 24 months, and marginal integrity.
- Secondary outcomes: postoperative sensitivity, aesthetic appearance, and secondary caries incidence (where reported).

Detailed domain-level RoB 2.0 assessments are provided in Supplementary Table S1. Most studies had low risk in randomization and intervention adherence domains, but three exhibited ‘some concerns’ related to outcome measurement blinding.

No statistical syntheses (e.g., meta-analyses) were conducted due to clinical and methodological heterogeneity across the included studies. These differences included variability in restoration techniques, materials used (e.g., bulk-fill type and viscosity), adhesive strategies, isolation methods, and outcome evaluation systems (e.g., USPHS, FDI, or modified indices). As a result, findings were presented descriptively and grouped by key clinical endpoints—retention, two-year survival, and marginal integrity. The robustness of the findings was explored narratively across methodological subgroups. For instance, outcomes were qualitatively compared across studies using different adhesive systems or cavity designs. No consistent superiority of one approach over another was identified across the studies.

Due to the small number of eligible studies per clinical outcome (typically fewer than 10 per subgroup), funnel plots were explored but could not be reliably interpreted. No visual evidence of significant asymmetry was noted, though the power to detect such bias was limited. Moreover, selective outcome reporting remains a possibility: secondary outcomes, such as aesthetics, secondary caries, or postoperative sensitivity, were not uniformly reported and often lacked consistent assessment tools. These limitations highlight the importance of standardized outcome definitions and reporting practices in future trials to improve data synthesis and reduce potential reporting bias.

### 3.2. Primary Clinical Outcomes

Retention outcomes were reported in most studies, predominantly using validated clinical criteria, such as USPHS or FDI systems [23,24,25,26,27]. Follow-up periods ranged from 12 to 60 months. Both bulk-fill and incremental composite restorations showed high short-term retention, often exceeding 90% at 12 months. One study found slightly higher early retention rates for bulk-fill materials, possibly due to their simpler placement and lower risk of voids or layering errors [11]. In contrast, another study found no statistically significant difference in retention between the two approaches [12]. Overall, bulk-fill composites appeared to perform as well as incremental techniques, making them a practical option in pediatric dentistry, especially when reduced chair time is needed.

Survival rate: Approximately half of the included studies evaluated clinical survival beyond 12 months, with several extending to 24 months. Across these trials, survival rates for both bulk-fill and incremental composite restorations were consistently high, typically above 85–90%, regardless of material or technique. Two studies reported notably higher survival rates for bulk-fill materials at 12–18 months, with values ranging from 93% to nearly 100% [12,19]. Similar outcomes were observed in other studies [11,15,17], which showed minimal or no difference in survival rates between techniques. Overall, the survival data supported the non-inferiority of bulk-fill composites in clinical performance, confirming their durability in pediatric patients over at least one to two years.

Marginal integrity: Most studies used USPHS or FDI criteria to assess marginal adaptation [25,26,27]. Overall, both bulk-fill and conventional composites demonstrated good clinical performance with minor deterioration over time. One trial found that conventional composites had less marginal discoloration, implying a small aesthetic advantage [12]. Conversely, another study reported significantly better Alpha scores for bulk-fill restorations (97%) compared to conventional techniques (66.7%) at 12 months [13]. Three additional studies found no meaningful difference in marginal adaptation between groups, with both materials performing at clinically acceptable levels [11,14,15]. Overall, operator skill, material flow, and cavity shape seem to matter more than whether you use bulk-fill or incremental layering.

### 3.3. Secondary Clinical Outcomes

Secondary clinical outcomes—including aesthetics, postoperative sensitivity, surface texture, anatomical form, and secondary caries—were less consistently reported across studies.

Table 2 summarizes the assessment methods, time points, and main findings for postoperative sensitivity, aesthetic evaluation, and secondary caries in the included studies.

Aesthetic performance, particularly color match, was deemed clinically acceptable in both groups in most trials. The study of Banon et al. (2024) found a statistically significant advantage for bulk-fill materials (ACTIVA) in color match (*p* = 0.002) [12], while others reported no significant differences between groups [14,17].

Postoperative sensitivity was evaluated in a limited number of studies. No significant differences were reported between groups in most cases. Notably, in one study (Öter et al., 2018), sensitivity was initially higher in the bulk-fill group at baseline but resolved by the six-month follow-up [8].

Regarding surface texture and anatomical form, studies generally found no clinically relevant differences. Two trials noted slightly better surface quality and anatomical form in the bulk-fill group [13,15], although the findings were not universally replicated.

The incidence of secondary caries was low across all studies. Several studies explicitly reported no cases during the follow-up period, supporting the cariostatic potential of both techniques when properly applied [8,11,12,17].

Overall, these results suggest that bulk-fill composites are comparable to incremental techniques in terms of secondary clinical outcomes, with no consistent evidence of superiority or increased risk in pediatric applications.

### 3.4. Procedural Outcomes

Several studies reported that bulk-fill composites save time during placement. Gindri et al. (2022) found that bulk-fill restorations took less time to place and were easier to handle [15]. Banon et al. (2024) confirmed these results, showing that bulk-fill placement reduced the operative time by about 2.4 min compared to incremental layering (*p* < 0.001) [12]. Most studies applied bulk-fill materials in a single increment of up to 4–5 mm, while conventional composites required multiple layers. Although not all studies measured total chair time, the simplified protocol for bulk-fill was a common advantage.

Ease of handling was assessed in a limited number of studies. Two studies [12,15] found that bulk-fill resins were easier to manipulate compared to conventional composites, citing reduced technique sensitivity and improved flow properties. However, other studies (e.g., [17]) observed no significant difference in ease of use between the two materials.

Overall, these findings support a favorable procedural profile for bulk-fill composites, especially in pediatric settings where time efficiency and operator convenience are critical factors.

### 3.5. Failure Characteristics

Mechanical failures, particularly retention loss and minor chipping, were reported in several studies involving bulk-fill materials. For instance, Deepika et al. (2022) observed retention loss at the occlusal surface between 6 and 12 months [13], while Ehlers et al. (2019) noted minor marginal chipping, likely due to mechanical stress [14].

In contrast, three studies reported no clinical failures in either the bulk-fill or conventional composite groups during their respective follow-up periods—namely, Akman and Tosun (2020), Sarapultseva and Sarapultsev (2019), and Öter et al. (2018) [8,11,22].

Olegário et al. (2022) highlighted isolation-related failures, reporting partial or complete restoration loss in the bulk-fill group between 12 and 24 months. This finding suggests that inadequate isolation (e.g., absence of rubber dam) may negatively impact long-term restoration success [21].

Additionally, Banon et al. (2024) identified marginal discoloration as the primary late-stage failure occurring between 18 and 24 months, pointing to issues with marginal adaptation in bulk-fill materials [12].

### 3.6. Time-Based Success Analysis

Success rates of restorations were consistently high across all reported time points, though slight variations emerged between techniques. At 6 and 12 months, bulk-fill and conventional composites performed similarly, with average success rates exceeding 90% in both groups. For example, at six months, five studies, including that of Deepika et al. (2022), reported 95.2% success for bulk-fill and 94.8% for conventional restorations [13]. At 12 months, success remained high, with bulk-fill slightly outperforming conventional techniques (91.7% vs. 89.2%). At 18 months, Massa et al. (2022) observed a more pronounced difference, favoring bulk-fill (76.8%) over conventional restorations (62.9%), possibly reflecting improved material stability or reduced technique sensitivity [19]. By 24 months, however, results became more variable. With that, Sarapultseva and Sarapultsev (2019) reported high success for both techniques, and conventional composites showed a numerically higher success rate (97.7%) compared to bulk-fill (82.4%) [22]. This may be attributed to cavity complexity, operator factors, or longer-term wear resistance.

Overall, these findings suggest that both techniques demonstrate high short-term success, with bulk-fill composites maintaining favorable performance through mid-term follow-up, though continued long-term monitoring is warranted.

### 3.7. Analysis by Cavity Classification

Several studies stratified outcomes based on cavity classification, underscoring how lesion complexity may influence restorative success. For Class I cavities, three studies, including that of Deepika et al. (2022), reported high success rates ranging from 85% to 100% for bulk-fill composites and 80% to 100% for conventional techniques [13]. No significant differences were observed, suggesting that both techniques were equally effective for simple occlusal restorations.

In contrast, for Class II cavities, which pose greater technique sensitivity due to proximal box involvement, outcomes were more variable. Two studies reported success rates for bulk-fill materials ranging from 76% to 93%, while conventional composites ranged from 62% to 95%. These mixed findings reflect variations in clinical protocols and operator experience, and no consistent technique superiority was established.

One study—by Gindri et al. (2022)—specifically analyzed multiple-surface cavities and found higher success rates for conventional composites (88–90%) compared to bulk-fill composites (75–93.7%) [15]. This result suggests that incremental layering may offer better marginal adaptation and durability in more complex lesions.

Collectively, the findings indicate that while bulk-fill composites performed comparably in less complex restorations, incremental techniques may still be preferred for multi-surface or Class II restorations due to their potential for enhanced adaptation in challenging anatomy.

### 3.8. Other Factors

Operator expertise emerged as a significant determinant of restoration success. In studies involving experienced clinicians, such as that of Gindri et al. (2022), success rates for both bulk-fill and conventional composites ranged from 90% to 100%, indicating that when restorations are placed by skilled practitioners, both techniques yield reliable outcomes [15]. In contrast, Massa et al. (2022) reported that less experienced operators were associated with significantly higher failure rates, with bulk-fill restorations exhibiting a 3.26× increased risk of failure (*p* = 0.001) [19]. Additionally, studies that involved multiple operators demonstrated greater variability in results, reinforcing the influence of technique consistency and protocol adherence. These findings underscore the importance of training and standardization, particularly when implementing newer materials in pediatric restorative procedures.

Although direct cost analyses were not uniformly performed, procedural efficiency and resource usage were discussed in several studies. Gindri et al. (2022) found that bulk-fill techniques reduced operative time by an average of 2.37 ± 0.63 min and required less material, both of which contribute to lower procedural costs [15]. Furthermore, bulk-fill composites were perceived as less technique-sensitive, a distinct advantage in high-turnover pediatric clinics where time and operator experience may vary [19].

However, in complex cases, some clinicians preferred incremental layering due to its greater control during placement and adaptability to irregular cavity anatomy. Overall, the findings support bulk-fill composites as a cost-efficient and clinically practical option, particularly beneficial in settings where treatment speed and simplification are prioritized.

### 3.9. Analysis of Systematic Reviews with a Relevant Review Question

This section synthesizes the findings of three systematic reviews—Amend et al., Schwendicke et al., and the present review—on the clinical performance of bulk-fill versus conventional composite restorations in primary teeth (Table 3) [23,24]. The analysis was structured around alignment with the PICO framework, methodological quality using AMSTAR-2 criteria, and relevance for clinical practice.

Amend et al. (2022) exclusively included primary teeth (29 RCTs), partially aligning with the PICO question [23]. However, it lacked direct subgroup analyses comparing bulk-fill and conventional composite materials and reported outcomes such as marginal adaptation only qualitatively. Furthermore, 86% of included studies were assessed as high risk of bias, limiting confidence in the conlusions. Schwendicke et al. (2016) employed a network meta-analysis but incorporated a mixed dentition dataset (only 8 of 36 studies focused on primary teeth) [24]. This compromised the pediatric specificity of their findings. Additionally, outcomes such as two-year survival were not analyzed as standalone metrics, and the included bulk-fill restorations were not consistently placed using bulk techniques [24]. In contrast, the present review demonstrated stronger alignment with the defined PICO elements and achieved a moderate AMSTAR-2 confidence rating. It included only in vivo RCTs in children aged 3–12 years with primary teeth, directly comparing bulk-fill and conventional composite techniques, and emphasized clinically meaningful endpoints—retention, two-year survival, and marginal integrity—using validated criteria (USPHS and FDI).

Across all three reviews, findings suggested comparable clinical performance between bulk-fill and conventional composite techniques. Both showed high short-term retention rates (>90% at 12 months) and similar two-year survival outcomes (~85–90%). The marginal integrity of restorations demonstrated mixed results: some studies noted slightly better adaptation with incremental techniques in complex cavities, while others found no significant differences. Notably, bulk-fill composites consistently offered time-saving advantages (2–4 min per restoration) without compromising survival or retention.

However, critical limitations persist. Neither Amend et al. nor Schwendicke et al. assessed two-year survival as a dedicated outcome, and marginal adaptation remains underreported or qualitatively assessed [23,24]. Moreover, heterogeneity in adhesive protocols, cavity classifications, operator technique, and outcome definitions constrained direct comparisons across studies. The present review also evaluated secondary outcomes, including postoperative sensitivity, aesthetic performance, and secondary caries incidence. These were inconsistently reported but showed no clinically significant differences between bulk-fill and conventional composite groups where data were available. Postoperative sensitivity was low across studies, and caries-related failures were infrequent. Future studies should prioritize standardization of methodology, particularly with respect to adhesive systems, isolation methods (e.g., rubber dam vs. cotton rolls), recall intervals, and scoring criteria. Greater attention should also be paid to patient-centered outcomes, such as comfort, aesthetic satisfaction, and procedural acceptability, in pediatric populations.

## 4. Discussion

This systematic review synthesized evidence from 13 randomized controlled trials evaluating the clinical performance of bulk-fill composite restorations versus traditional incremental layering techniques in primary teeth. The primary outcomes—retention, two-year survival, and marginal integrity—were supplemented by secondary measures, including aesthetic outcomes, postoperative sensitivity, and restoration failure characteristics. Together, these outcomes provide a clinically meaningful foundation for optimizing restorative strategies in pediatric dentistry.

The evidence supports the conclusion that bulk-fill composites perform comparably to incremental composites in terms of short- to mid-term retention and survival. Studies such as those of Deepika et al. (2022), Massa et al. (2022), and Sarapultseva and Sarapultsev (2019) [13,19,22] demonstrated similar success rates between techniques, highlighting the operational advantages of bulk-fill materials in terms of placement speed, simplicity, and reliability over time—factors especially valuable in pediatric settings, where patient cooperation may be limited.

Marginal integrity, an essential factor in preventing microleakage and secondary caries, was also consistently high across both materials. Although one study reported marginally better outcomes with incremental layering [17], the majority—including Banon et al. (2024), Lucchi et al. (2024), and Gindri et al. (2022)—found no significant differences [12,15,18]. These findings are supported by larger trials using standardized assessment systems, such as USPHS and FDI, which have shown no consistent clinical advantage for either method in marginal adaptation [25,26].

Secondary outcomes, such as postoperative sensitivity and aesthetic performance, were infrequently reported but generally supported the non-inferiority of bulk-fill techniques. Minimal or no sensitivity was noted in both groups by Akman and Tosun (2020) and Massa et al. (2022) [11,19]. Aesthetic performance, although more relevant in anterior restorations, was deemed clinically acceptable in posterior teeth by Akman and Tosun (2020), Gindri et al. (2022), and Cantekin and Gumus (2014) [11,15,16]. These results align with the FDI framework’s criteria for clinical acceptability, supporting the conclusion that bulk-fill composites provide aesthetically satisfactory outcomes for posterior pediatric restorations [27].

Notably, failure patterns differed based on restoration type. While overall failure rates were low, bulk-fill restorations were more prone to early retention loss or chipping, especially in studies involving less experienced operators [13,14,19]. Conversely, incremental techniques showed slightly better performance in complex Class II or multi-surface cavities, where layered placement may offer superior control [15]. These observations underscore the need to match the restorative strategy to cavity complexity and operator experience.

A time-based analysis of clinical success revealed high short-term effectiveness for both techniques (success rates > 90% at 6–12 months). However, some divergence emerged at 24 months, particularly in studies with compromised isolation or involving multiple operators [12,21]. These observations suggest that bulk-fill materials maintain high short-term effectiveness, but careful technique and follow-up remain essential for long-term success.

The methodological quality of the included studies was generally acceptable, with most rated as low risk of bias across randomization and intervention adherence domains. However, three studies were judged to have “some concerns” in blinding and outcome measurement domains. Detailed domain-level RoB 2.0 assessments are provided in Supplementary Table S1. This variation in methodological rigor necessitates cautious interpretation of secondary outcome data.

Several key limitations of the evidence base must be acknowledged:

- There was substantial heterogeneity in materials used (e.g., viscosity of bulk-fill), adhesive protocols, and evaluation criteria.
- Follow-up durations varied from 12 to 60 months, with relatively few studies extending beyond 2 years.
- The use of different outcome scoring systems (USPHS, FDI, or modified criteria) complicated cross-study comparisons [25,26].
- Some studies paired bulk-fill and incremental composites with different adhesive systems, introducing potential confounding.
- Although a split-mouth design was employed in several studies, the possibility of cross-arch effects or intraoral variability may have influenced results.
- No meta-analysis was performed due to clinical and methodological heterogeneity, in accordance with PRISMA 2020 guidance.

The GRADE approach indicated moderate certainty of evidence for retention and survival outcomes, but low certainty for secondary measures due to inconsistent reporting and small sample sizes. Moreover, funnel plot asymmetry could not be reliably interpreted because most outcomes were reported in fewer than 10 studies.

Despite these limitations, this review adhered to a registered protocol, followed PRISMA 2020 guidelines, and incorporated independent RoB 2.0 assessments. While inter-rater reliability metrics (e.g., Cohen’s kappa) were not formally calculated, all screening and extraction discrepancies were resolved through consensus, ensuring methodological transparency.

From a clinical standpoint, bulk-fill composites appear to be a pragmatic and non-inferior alternative to incremental techniques for restoring carious primary teeth. They are particularly advantageous in cases requiring efficient, minimally stressful treatment sessions. A hybrid technique—combining a flowable bulk-fill base with an incremental occlusal layer—may offer an optimal balance between speed and polymerization stress control. Additionally, strict rubber dam isolation, consistent operator training, and clearly defined recall intervals should be emphasized in future trials [21].

To contextualize this review, prior systematic reviews were appraised using the AMSTAR-2 tool. Many lacked protocol registration, formal bias assessments, or clearly defined PICO frameworks. For example, the study by Amend et al. (2022) was rated as high quality, while others were methodologically limited [23]. The findings of this review are also supported by broader meta-analyses in permanent teeth, such as that of Veloso et al. (2019), which found no significant performance difference between bulk-fill and incremental composites in posterior restorations [1].

In summary, bulk-fill composites represent a clinically effective and time-efficient option for pediatric restorative care, especially when used in appropriate clinical contexts and with adherence to standardized protocols. Future trials should prioritize methodological rigor, outcome standardization, and operator-level reporting to further inform evidence-based practice.

## 5. Conclusions

This systematic review supported the clinical efficacy and practical utility of bulk-fill composite restorations as a viable and reliable alternative to conventional incremental layering techniques in primary teeth. Across 13 in vivo randomized controlled trials, bulk-fill materials demonstrated comparable performance in terms of retention, 2-year survival, marginal integrity, and secondary outcomes, with no evidence of increased clinical risk.

Despite these encouraging findings, several methodological and reporting limitations were identified. Future research should adopt standardized evaluation criteria (e.g., USPHS or FDI), apply uniform follow-up durations, and ensure comprehensive outcome reporting to improve cross-study comparability. Key evidence gaps included the lack of long-term studies beyond 24 months, insufficient data on cavity-type-specific outcomes, and limited formal assessments of cost-effectiveness.

From a clinical perspective, bulk-fill composites offer notable advantages in placement speed and technique simplicity, both of which are particularly beneficial for treating young or uncooperative patients. However, these advantages depend on strict adherence to isolation protocols and sufficient operator training.

Future studies should focus on:

- Detailed analysis of failure modes and timing.
- Standardized documentation of composite and adhesive materials used.
- Systematic assessment of technique sensitivity across different operator skill levels.
- Transparent reporting of operator experience and training, ideally following CONSORT guidelines.

Addressing these priorities through well-designed, multicenter randomized trials will enhance the quality of evidence and refine restorative protocols—ultimately improving treatment outcomes in pediatric dental practice.

## Figures and Tables

**Table 2:** Summary of secondary outcomes’ reporting by study.

Study (First Author, Year)	Postoperative Sensitivity	Aesthetic Evaluation	Secondary Caries
Akman, 2020 [11]	Assessed at baseline, 3, 6, and 12 months using modified USPHS criteria. No significant difference between materials; all received Alpha scores at all time points.	Evaluated at same intervals using modified USPHS criteria (color match, marginal discoloration, anatomical form). No significant differences; 100% Alpha scores for color match and anatomical form; Equia showed poorer marginal adaptation at 6 and 12 months.	Assessed at all intervals with modified USPHS criteria; 100% success for all groups, no secondary caries detected radiographically.
Banon, 2024 [12]	Assessed every 6 months up to 24 months using USPHS-Ryge criteria. No significant differences between materials.	Color match and marginal discoloration assessed every 6 months using USPHS-Ryge. ACTIVA had better color match (*p* = 0.002), but worse marginal discoloration (*p* = 0.0143); Dyract showed more color changes over 24 months.	Radiographic evaluation every 12 months; no significant difference between groups. Three Dyract and four ACTIVA restorations developed secondary caries.
Deepika, 2022 [13]	Evaluated immediately post-op, at 6 and 12 months. No sensitivity at 12 months; one case at 6 months.	Color match, marginal discoloration, marginal integrity, and anatomic form assessed using modified USPHS. At 12 months, significant differences in marginal discoloration (*p* = 0.04), marginal integrity (*p* < 0.001), and anatomic form (*p* = 0.02), favoring bioactive resin-modified glass ionomer.	Not explicitly listed; no complications (pain, swelling, fistula) at 12 months.
Ehlers, 2019 [14]	Baseline and 1 year using FDI criteria. No severe postoperative sensitivities or side effects; both materials clinically excellent.	Baseline and 1 year, FDI criteria (surface luster, staining, color match, translucency, anatomical form). Compomer had better color match and translucency; both materials clinically excellent.	Baseline and 1 year, FDI criteria (recurrence of caries). No secondary caries observed.
Gindri, 2022 [15]	Not specifically reported; FDI criteria include biological properties, but postoperative sensitivity was not singled out or discussed as a separate finding.	Assessed at baseline, 6, and 12 months using FDI criteria: surface gloss, surface staining, marginal staining, and anatomical form. No restoration required intervention due to aesthetic parameters; only minor changes observed over 12 months.	Evaluated at baseline, 6, and 12 months using FDI criteria (recurrence of caries). Only one restoration in each group failed due to recurrence of caries after one year; overall, secondary caries was rare.
Cantekin, 2014 [16]	Monitored via clinical history of pain reported by parents and children; no pain or symptoms reported at 6 and 12 months; not directly evaluated with scoring system.	Not specifically defined or quantified; aesthetic restorations mentioned but no detailed criteria or results provided.	Evaluated clinically and radiographically for pathological changes and radiolucency; 100% clinical success with no secondary caries at 6 and 12 months.
Lardani, 2022 [17]	3, 6, and 12 months using FDI criteria (biological properties). Slight decrease in “clinically excellent” over time; no significant difference between materials or cavity classes (*p* = 0.16).	3, 6, and 12 months, FDI criteria (surface luster, staining, color stability, translucency, anatomic form). Both materials worsened over time; no significant difference (*p* = 0.19–1).	3, 6, and 12 months, FDI criteria. Slight decrease in “clinically excellent” restorations; no significant difference between groups. Failure rate at 12 months: 2.2% for both.
Lucchi, 2024 [18]	Not assessed long term; sensitivity excluded, as unreliable in pediatric patients.	Color and translucency not considered; marginal dyschromia evaluated visually and with mirror using modified USPHS. Lower incidence in second molars; after 5 years, 37% rated Bravo for superficial marginal dyschromia.	Visual and radiographic assessment with modified USPHS; lower incidence in second molars. After 5 years, 14% of first molars and 13% of second molars had secondary caries.
Massa, 2022 [19]	Not reported in the provided data.	Not reported in the provided data.	Not reported in the provided data.
Olegário, 2022 (Pulpectomy) [20]	Not directly evaluated as a clinical restoration parameter; parent/child acceptance questionnaire used; child self-assessment with Wong–Baker Faces Pain Scale immediately after treatment; no differences found between groups.	Not evaluated as a clinical restoration parameter; acceptance of appearance assessed by questionnaire; both stainless-steel crowns and bulk-fill composites well accepted by children and parents with no significant difference.	Included as a criterion for restoration success; failures in bulk-fill group related to bulk fracture leading to bacterial infiltration; secondary caries noted in dentin in isolation study; follow-up up to 24 months.
Olegário, 2022 (Isolation) [21]	Self-reported pain immediately after treatment (Wong–Baker Faces Pain Scale). No significant difference between groups.	Not a primary outcome; focus on restoration survival, cost, and patient behavior.	Secondary caries in dentine accounted for 25.37% of failures, after bulk fracture (52.24%).
Sarapultseva, 2019 [22]	Baseline, 6, 18, 24 months using modified Ryge criteria. No postoperative sensitivity at any point.	Color match, marginal discoloration, surface texture (modified Ryge). No significant differences; two Bravo scores for marginal discoloration in each group.	Baseline, 6, 18, 24 months using modified Ryge. No secondary caries in any restoration; minor difference (3.7%) at 24 months.
Oter, 2018 [8]	Baseline, 6 months, 1 year using modified USPHS. Sensitivity higher in bulk-fill at baseline (*p* < 0.05), resolved by 6 months; no difference at 6 or 12 months.	Color match, marginal discoloration, surface texture, anatomic form (modified USPHS). No significant differences at any interval. Bravo scores for marginal discoloration increased over time in both groups.	Baseline, 6 months, 1 year using modified USPHS. No secondary caries in any group at any time point.

**Table 3:** Summary of systematic reviews aligned with the PICO framework and AMSTAR-2 quality ratings.

Study Type	Focus	Population	Interventions	Comparator	Outcomes Measured	Follow-Up	AMSTAR-2 Rating	Ref.
Systematic Review	Bulk-fill vs. conventional composites	Primary teeth only	Bulk-fill composites	Conventional composites	Retention, survival, marginal adaptation (qualitative)	12–84 months	Low	[23]
Network Meta-Analysis	Mixed dentition (primary + permanent teeth)	8/36 studies in primary teeth	Bulk-fill composites	Conventional composites	Survival, failure modes	24–48 months	Critically Low	[24]
Systematic Review	Bulk-fill vs. incremental composites in children	Pediatric, primary teeth only	Bulk-fill composites	Incremental composites	Retention, 2-year survival, marginal integrity, others	12–60 months	Moderate	Present

## Data Availability

This study was based on a systematic review of previously published data. No new experimental data were generated or analyzed. Extracted data supporting the findings of this review are available from the corresponding author upon reasonable request.

## Supplementary Materials

The following supporting information can be downloaded at: https://www.mdpi.com/article/10.3390/dj13070320/s1, Supplementary S1: Full Search Strategies; Table S1: Risk of Bias Assessment Results.
