# Supplementary material for: Clinical Performance of Bulk-Fill Versus Incremental Composite Restorations in Primary Teeth: A Systematic Review of In Vivo Evidence
Source: Dent J (Basel). 2025 Jul 15;13(7):320. doi: 10.3390/dj13070320 (PMC12294032; doi:10.3390/dj13070320)
Supplement: Supplementary file 1 [file dentistry-13-00320-s001.zip › Supplementary_Search_Strategies.pdf]

Supplementary Appendix: Full Search Strategies

This appendix provides the complete database-specific search strategies used in the systematic review titled 'Clinical Performance of Bulk-Fill Versus Incremental Composite Restorations in Primary Teeth: A Systematic Review'. The searches were conducted on March 25, 2025, across PubMed and Scopus.

1. PubMed Search Strategy

Date searched: March 25, 2025

Filters applied:

- Language: English
- Species: Humans
- Study type: Clinical Trial, Randomized Controlled Trial

Search string:

> ("bulk-fill composite"[All Fields] OR "bulk fill composite"[All Fields] OR "bulk-fill resin composite"[All Fields]) AND ("pediatric dentistry"[MeSH Terms] OR "primary teeth"[MeSH Terms] OR "child"[MeSH Terms]) AND ("restoration"[All Fields] OR "filling"[All Fields] OR "composite"[All Fields]) AND ("retention"[All Fields] OR "survival"[All Fields] OR "marginal integrity"[All Fields])

Note: No age filters were applied in PubMed to avoid excluding relevant studies involving children under 6 years. Pediatric relevance (ages 3–12) was confirmed during full-text screening.

> "Bulk-Fill"[All Fields] AND ("composite"[All Fields] OR "composite s"[All Fields] OR "composited"[All Fields] OR "composites"[All Fields] OR "compositing"[All Fields] OR "composition"[All Fields] OR "compositional"[All Fields] OR "compositions"[All Fields]) AND ("restorability"[All Fields] OR "restorable"[All Fields] OR "restorated"[All Fields] OR "restoration"[All Fields] OR "restoration s"[All Fields] OR "restorations"[All Fields] OR "restorative"[All Fields] OR "restoratives"[All Fields] OR "restore"[All Fields] OR "restored"[All Fields] OR "restores"[All Fields] OR "restoring"[All Fields]) AND ("tooth, deciduous"[MeSH Terms] OR ("tooth"[All Fields] AND "deciduous"[All Fields]) OR "deciduous tooth"[All Fields] OR ("primary"[All Fields] AND "teeth"[All Fields]) OR "primary teeth"[All Fields])

Additional advanced PubMed search string used:

2. Scopus Search Strategy

Date searched: March 25, 2025

Filters applied:

- Language: English
- Document type: Article
- Subject area: Dentistry

Search string:

TITLE-ABS-KEY("bulk-fill composite" OR "bulk fill composite" OR "bulk-fill resin composite") AND TITLE-ABS-KEY("pediatric dentistry" OR "primary teeth" OR "child") AND TITLE-ABS-KEY("restoration" OR "filling" OR "composite") AND TITLE-ABS-KEY("retention" OR "survival" OR "marginal integrity")

Note: No age restrictions were set during the database search. Eligibility was determined during screening, focusing on studies involving children aged 3 to 12 years.
