# Supplementary material for: Clinical Performance of Bulk-Fill Versus Incremental Composite Restorations in Primary Teeth: A Systematic Review of In Vivo Evidence
Source: Dent J (Basel). 2025 Jul 15;13(7):320. doi: 10.3390/dj13070320 (PMC12294032; doi:10.3390/dj13070320)
Supplement: Supplementary file 1 [file dentistry-13-00320-s001.zip › Table_supl.pdf]

Table S1. Risk of Bias Assessment Results

Study	Bias: Randomiz	Bias: Interven	Bias: Miss Data	Bias: Outcome Measur	Bias: Report	Study Design	Sample Size	Follow-up Period	Evaluation Crit	Population Det	Intervention Mat	Ref
Akman and Tosun, 2020	Low risk	Low risk	Low risk	Low risk	Low risk	RCT, Prospective	30 patients, 160 restorations	12 months	Modified USPHS	Children aged 6–10 years, involving primary molars	Bulk-fill: Sonicfill and X-tra fil; Conventional: Filtek Z550	Akman and Tosun
Banon et al., 2024	Low risk	Low risk	Low risk	Low risk	Low risk	RCT, Split-mouth	20 children, 96 molars	24 months	USPHS-Ryge	Children aged 5–10 years, involving primary molars	Bulk-fill: ACTIVA BioACTIVE; Conventional: Dyract eXtra	Banon et al.
Deepika et al., 2022	Some concerns	Low risk	Low risk	Low risk	Low risk	RCT, Split-mouth	50 children, 100 primary molars	12 months	Modified USPHS	Children aged 5–9 years, involving maxillary and mandibular primary molars	Bulk-fill: ACTIVA Bioactive restorative; Conventional: Beautifil flow Plus F00	Deepika et al.
Ehlers et al., 2019	Some concerns	Some concerns	Low risk	Some concerns	Some concerns	RCT, Split-mouth	32 children	12 months	FDI	Children aged 4–9 years, involving	Bulk-fill: Venus Bulk Fill;	Ehlers et al.

Study	Bias: Randomiz	Bias: Interven	Bias: Miss Data	Bias: Outcome Measur	Bias: Report	Study Design	Sample Size	Follow-up Period	Evaluation Crit	Population Det	Intervention Mat	Ref
										primary molars	Conventional: Dyract eXtra	
Gindri et al., 2022	Low risk	Low risk	Low risk	Low risk	Low risk	RCT	65 participants, 140 restorations	12 months	FDI	Children aged 5.2–8.2 years, involving primary molars	Bulk-fill: Filtek Bulk Fill; Conventional: Filtek Z350 XT	Gindri et al.
Cantekin and Gumus, 2014	Some concerns	Low risk	Low risk	Low risk	Low risk	RCT, Prospective	20 children	12 months	Modified Zurn and Seale	Children aged 5–7 years, involving maxillary and mandibular primary molars	Bulk-fill: SDR flowable composite; Conventional: Aelite LS Posterior	Gumus and Cantekin
Lardani et al., 2022	Some concerns	Low risk	Low risk	Low risk	Low risk	Split-mouth RCT	45 children	12 months	FDI	Children aged 5–9 years, involving primary first and second molars	Bulk-fill: ACTIVA BioActive and SDR Bulk-fill	Lardani et al.

Study	Bias: Randomiz	Bias: Interven	Bias: Miss Data	Bias: Outcome Measur	Bias: Report	Study Design	Sample Size	Follow-up Period	Evaluation Crit	Population Det	Intervention Mat	Ref
Lucchi et al., 2024	Some concerns	Low risk	Low risk	Low risk	Low risk	Retrospective study	198 patients (88 males, 110 females)	5 years	USPHS (United States Public Health Service)	Children aged 0-12 years, with 673 restorations on primary molars	Bulk-fill composite (Filtek Bulk-Fill Flow, 3M ESPE)	Lucchi et al., 2024
Massa et al., 2022	Some concerns	Low risk	Low risk	Low risk	Low risk	RCT	62 subjects, 144 primary molars	18 months	FDI	Children aged 4.2–7.6 years, involving primary molars	Bulk-fill: Filtek Bulk Fill Posterior Restorative	Massa et al.
Olegário et al., 2021 (1)	Low risk	Low risk	Low risk	Low risk	Low risk	RCT	91 children	12 months	Roeleveld	Children aged 3–8 years, involving primary molars	Bulk-fill: Filtek Bulk Fill	Olegário et al.
Olegário et al., 2021 (2)	Low risk	Low risk	Low risk	Low risk	Low risk	RCT	93 children	24 months	Roeleveld	Children aged 4–8 years, involving primary molars	Bulk-fill: Filtek Bulk Fill composite resin (3M ESPE)	Olegário et al.

Study	Bias: Randomiz	Bias: Interven	Bias: Miss Data	Bias: Outcome Measur	Bias: Report	Study Design	Sample Size	Follow-up Period	Evaluation Crit	Population Det	Intervention Mat	Ref
Sarapultseva and Sarapultsev, 2019	Some concerns	Low risk	Low risk	Low risk	Low risk	Split-mouth RCT	27 children	24 months	Modified Ryge	Children aged 3–6 years, involving mandibular second primary molars	Bulk-fill: SDR (Dentsply); Conventional: Ceram-X mono (Dentsply)	Sarapultseva and Sarapultsev
Öter et al.	Some concerns	Low risk	Low risk	Low risk	Low risk	RCT, Split-mouth	80 children	12 months	Modified USPHS	Children aged 5.61–9.21 years, involving primary molars	Bulk-fill: Filtek Bulk-Fill Restorative; Conventional: Filtek Z250	Öter et al.

References

1. Akman, H.; Tosun, G. Clinical Evaluation of Bulk-Fill Resins and Glass Ionomer Restorative Materials: A 1-Year Follow-up Randomized Clinical Trial in Children. Niger J Clin Pract 2020, 23, 489–497, doi:10.4103/njcp.njcp_519_19.
2. Banon, R.; Vandenbulcke, J.; Van Acker, J.; Martens, L.; De Coster, P.; Rajasekharan, S. Two-Year Clinical and Radiographic Evaluation of ACTIVA BioACTIVE versus Compomer (Dyract® eXtra) in the Restoration of Class-2 Cavities of Primary Molars: A Non-Inferior Split-Mouth Randomised Clinical Trial. BMC Oral Health 2024, 24, 437, doi:10.1186/s12903-024-04132-w.
3. Deepika, U.; Sahoo, P.K.; Dash, J.K.; Baliarsingh, R.R.; Ray, P.; Sharma, G. Clinical Evaluation of Bioactive Resin-Modified Glass Ionomer and Giomer in Restoring Primary Molars: A Randomized, Parallel-Group, and Split-Mouth Controlled Clinical Study. J Indian Soc Pedod Prev Dent 2022, 40, 288–296, doi:10.4103/jisppd.jisppd_139_22.
4. Ehlers, V.; Gran, K.; Callaway, A.; Azrak, B.; Ernst, C.-P. One-Year Clinical Performance of Flowable Bulk-Fill Composite vs Conventional Compomer Restorations in Primary Molars. J Adhes Dent 2019, 21, 247–254, doi:10.3290/j.jad.a42519.
5. Gindri, L.D.; Cassol, I.P.; Fröhlich, T.T.; Rocha, R. de O. One-Year Clinical Evaluation of Class II Bulk-Fill Restorations in Primary Molars: A Randomized Clinical Trial. Braz Dent J 2022, 33, 110–120, doi:10.1590/0103-6440202205069.

6. Cantekin, K.; Gumus, H. In Vitro and Clinical Outcome of Sandwich Restorations with a Bulk-Fill Flowable Composite Liner for Pulpotomized Primary Teeth. J Clin Pediatr Dent 2014, 38, 349–354, doi:10.17796/jcpd.38.4.f718041225w7621q.
7. Lardani, L.; Derchi, G.; Marchio, V.; Carli, E. One-Year Clinical Performance of Activa™ Bioactive-Restorative Composite in Primary Molars. Children (Basel) 2022, 9, 433, doi:10.3390/children9030433.
8. Lucchi, P.; Mazzoleni, S.; Parcianello, R.G.; Gatto, R.; Gracco, A.; Stellini, E.; Ludovichetti, F.S. Bulk-Flow Composites in Paediatric Dentistry: Long Term Survival of Posterior Restorations. A Retrospective Study. J Clin Pediatr Dent 2024, 48, 108–114, doi:10.22514/jocpd.2024.084.
9. Massa, M.G.; Trentin, G.A.; Noal, F.C.; Franzon, R.; Lenzi, T.L.; de Araujo, F.B. Use of Bulk Fill Resin Composite and Universal Adhesive for Restoring Primary Teeth after Selective Carious Tissue Removal to Soft Dentin: A Randomized Clinical Trial. Am J Dent 2022, 35, 97–102.
10. Olegário, I.C.; Bresolin, C.R.; Pássaro, A.L.; de Araujo, M.P.; Hesse, D.; Mendes, F.M.; Raggio, D.P. Stainless Steel Crown vs Bulk Fill Composites for the Restoration of Primary Molars Post-Pulpectomy: 1-Year Survival and Acceptance Results of a Randomized Clinical Trial. Int J Paediatr Dent 2022, 32, 11–21, doi:10.1111/ipd.12785.
11. Olegário, I.C.; Moro, B.L.P.; Tedesco, T.K.; Freitas, R.D.; Pássaro, A.L.; Garbim, J.R.; Oliveira, R.; Mendes, F.M.; Raggio, D.P. Use of Rubber Dam versus Cotton Roll Isolation on Composite Resin Restorations' Survival in Primary Molars: 2-Year Results from a Non-Inferiority Clinical Trial. BMC Oral Health 2022, 22, 440, doi:10.1186/s12903-022-02449-y.
12. Sarapultseva, M.; Sarapultsev, A. Flowable Bulk-Fill Materials Compared to Nano Ceramic Composites for Class I Cavities Restorations in Primary Molars: A Two-Year Prospective Case-Control Study. Dent J (Basel) 2019, 7, 94, doi:10.3390/dj7040094.
13. Oter, B.; Deniz, K.; Cehreli, S.B. Preliminary Data on Clinical Performance of Bulk-Fill Restorations in Primary Molars. Niger J Clin Pract 2018, 21, 1484–1491, doi:10.4103/njcp.njcp_151_18.

1. .
